# Taxonomy and physiological characterisation of *Scheffersomyces titanus* sp. nov., a new D-xylose-fermenting yeast species from China

**DOI:** 10.1038/srep32181

**Published:** 2016-08-25

**Authors:** Xiao-Jing Liu, Wan-Nan Cao, Yong-Cheng Ren, Long-Long Xu, Ze-Hao Yi, Zheng Liu, Feng-Li Hui

**Affiliations:** 1 School of Life Science and Technology, Nanyang Normal University, Nanyang 473061, P. R. China; 2 Henan Provincial Key Laboratory of Funiu Mountain Insect Biology, Nanyang Normal University, Nanyang 473061, P. R. China

## Abstract

Three strains of a d-xylose-fermenting yeast species were isolated from the host beetle *Dorcus titanus* collected from two different localities in Henan Province, Central China. These strains formed two hat-shaped ascospores in conjugated and deliquescent asci. Multilocus phylogenetic analysis that included the nearly complete small subunit (SSU), the internal transcribed spacer (ITS) region and the D1/D2 domains of the large subunit (LSU) rDNAs, as well as RNA polymerase II largest subunit (*RPB1*) gene demonstrated that these strains represent a novel yeast species belonging to the genus *Scheffersomyces*. The phylogenetic analysis based on the nucleotide sequences of the xylose reductase (*XYL1*) gene supported the view that the new strains could be grouped as a unique species. Although this new species is highly similar to *Scheffersomyces stipitis*-like yeasts in terms of nrDNA sequences and morphological and physiological characteristics, the species can be clearly differentiated from its close relatives on the basis of the sequences of *XYL1* and *RPB1*. Therefore, a novel yeast species, *Scheffersomyces titanus* sp. nov., is proposed to accommodate these strains. The type strain is NYNU 14712^T^ (CICC 33061^T^ = CBS 13926^T^).

The genus *Scheffersomyces* was first proposed by Kurtzman and Suzuki (2010) with the description of *Scheffersomyces stipitis*, *S*. *segobiensis* and *S*. *spartiniae*, which were formerly assigned to the genus *Pichia*^1^,^2^. The genus was later expanded by the inclusion of seven related *Candida* species as new combinations and by three novel species, namely, *S*. *illinoinensis*, *S*. *quercinus* and *S*. *virginianus*, which have been isolated from rotten wood^3^. This expansion resulted from a detailed multilocus phylogenetic analysis that included the traditional SSU and LSU markers, the orthologous *RPB1* and the recently proposed ITS barcoding region for fungi^3^,^4^. Since then, the number of species belonging to this genus has continuously increased because additional species, including *S*. *cryptocercus*^5^, *S*. *parashehatae* and *S*. *xylosifermentans*^6^, and *S. henanensis*^7^, have been discovered and described.

The genus *Scheffersomyces* currently comprises 18 recognised yeast species^1^,^3^,^5^,^6^,^7^. Most of the species, including *S*. *cryptocercus*, *S. henanensis*, *S*. *illinoinensis*, *S*. *insectosa*, *S*. *lignosus*, *S*. *parashehatae*, *S*. *quercinus*, *S*. *segobiensis*, *S*. *shehatae*, *S*. *stipitis*, *S*. *xylosifermentans* and *S*. *virginianus*, possess rare ability to ferment d-xylose to ethanol, potentially allowing industrial utilization of this pentose from hemicellulosic plant residues^8^,^9^,^10^. *S*. *shehatae* and *S*. *stipitis* are considered as the most efficient ethanol producers among these naturally d-xylose-fermenting yeasts^9^,^11^. Despite the existence of these microorganisms, the efficiency and rates of d-xylose fermentation are low. As such, the feasibility of industrial production of lignocellulosic bioethanol remains a challenge^12^,^13^. Therefore, there is still a need for new yeasts capable of efficient d-xylose fermentation for bioethanol production.

During an investigation of the yeast community associated with insects obtained from central China, we isolated three d-xylose-fermenting yeasts whose physiological traits and ascospore morphology typically resembled those of the genus *Scheffersomyces*. Molecular phylogenetic data indicated that these strains represent a novel species closely related to *S*. *stipitis*-like yeasts. In this study, we describe this new species as *S. titanus* sp. nov.

## Results

### Phylogenetic analysis

**Figure 1:**
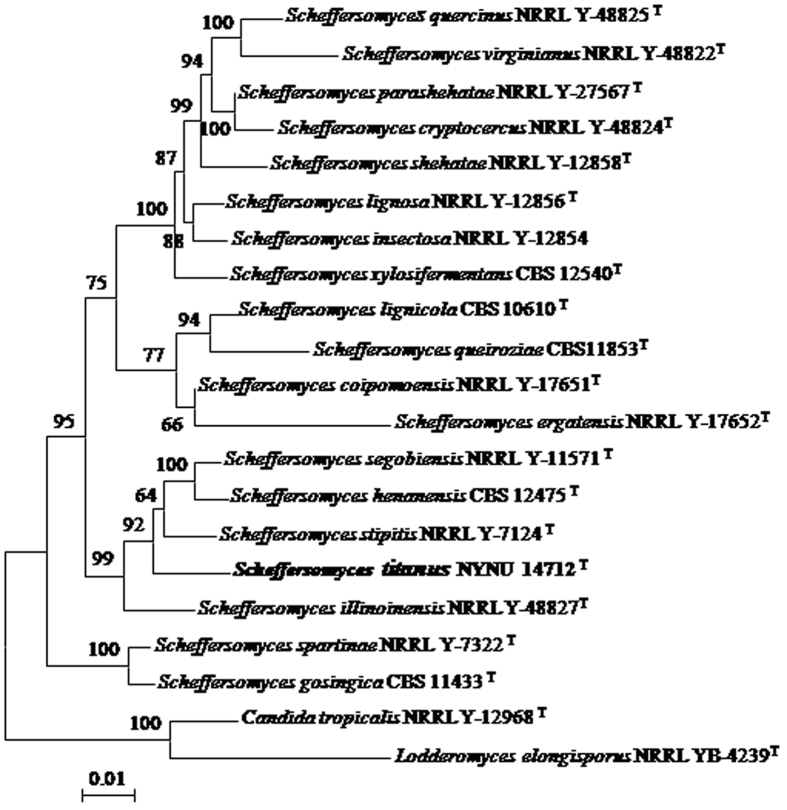
Phylogenetic tree constructed from the neighbour-joining analysis of the combined sequences of SSU, ITS, D1/D2 LUS and *RPB1*, depicting the relationships of *Scheffersomyces titanus* sp. nov. with closely related taxa in the *Scheffersomyces* clade. *Candida tropicalis* NRRL Y-12968^T^ was used as an outgroup taxon. Bootstrap percentages over 50% from 1000 bootstrap replicates are shown. Bar, 0.01 substitutions per nucleotide position.

**Figure 2:**
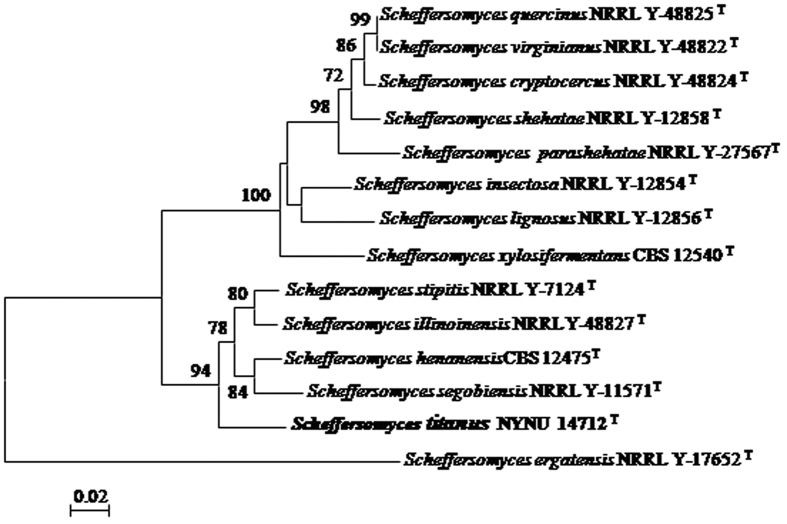
Phylogenetic tree reconstructed from the neighbour-joining analysis of *XYL1* sequences, depicting the relationships of *Scheffersomyces titanus* sp. nov. with closely related taxa in *S*. *stipitis* subclade. *Scheffersomyces ergatensis* NRRL Y-17652^T^ was used as an outgroup taxon. The numbers above each branch refer to bootstrap values out of 1000 repetitions. Bar, 0.02 substitutions per nucleotide position.

The sequences of nuclear rDNAs, including SSU, ITS and LSU, of the three strains of the new species were identical. This new species is closely related to the d-xylose-fermenting *Scheffersomyces* species, especially to *S. stipitis* and its close relatives, including *S*. *henanensis*, *S. illinoinensis* and *S*. *segobiensis* (Figs 1 and 2). The novel species differ from other four d-xylose-fermenting species in *S*. *stipitis* subclade by only 3 to 5 substitutions in the ITS region and the D1/D2 domain. As such, the species cannot easily be distinguished from other species in *S*. *stipitis* subclade by nDNA sequence analysis alone. The sequence of the easily amplified *XYL1* has been used to recognise cryptic species in the *Scheffersomyces* clade^3^,^5^,^6^,^7^. In the *XYL1* locus, the species differed significantly from those of other four *Scheffersomyces* species in the subclade by 5.6–7% sequence divergence (34–40 substitutions). This new species also differed from other four yeast species in the *S*. *stipitis* subclade by 11–12.5% sequence divergence (76–83 substitutions) in *RPB1*. These results showed that these strains are distinct from those of other species in this subclade.

Phylogenetic analysis was performed using the combined SSU, ITS, LSU and *RPB1* sequences, and *XYL1* gene sequences. In the multilocus phylogenetic tree, our strains clustered within the *S*. *stipitis* subclade as a separate branch with 92% bootstrap support (Fig. 1). The tree topology is in agreement with topologies obtained previously^3^,^5^,^6^,^7^. In the tree obtained with *XYL1* gene sequences (Fig. 2), the novel strains were placed in a well supported branch basal to the four species of *S*. *stipitis* subclade which were similar to those obtained from the multilocus phylogenetic tree. Results of these analyses confirmed that the three strains represent a distinct taxa of the genus *Scheffersomyces*.

### Morphology and Physiology

**Figure 3:**
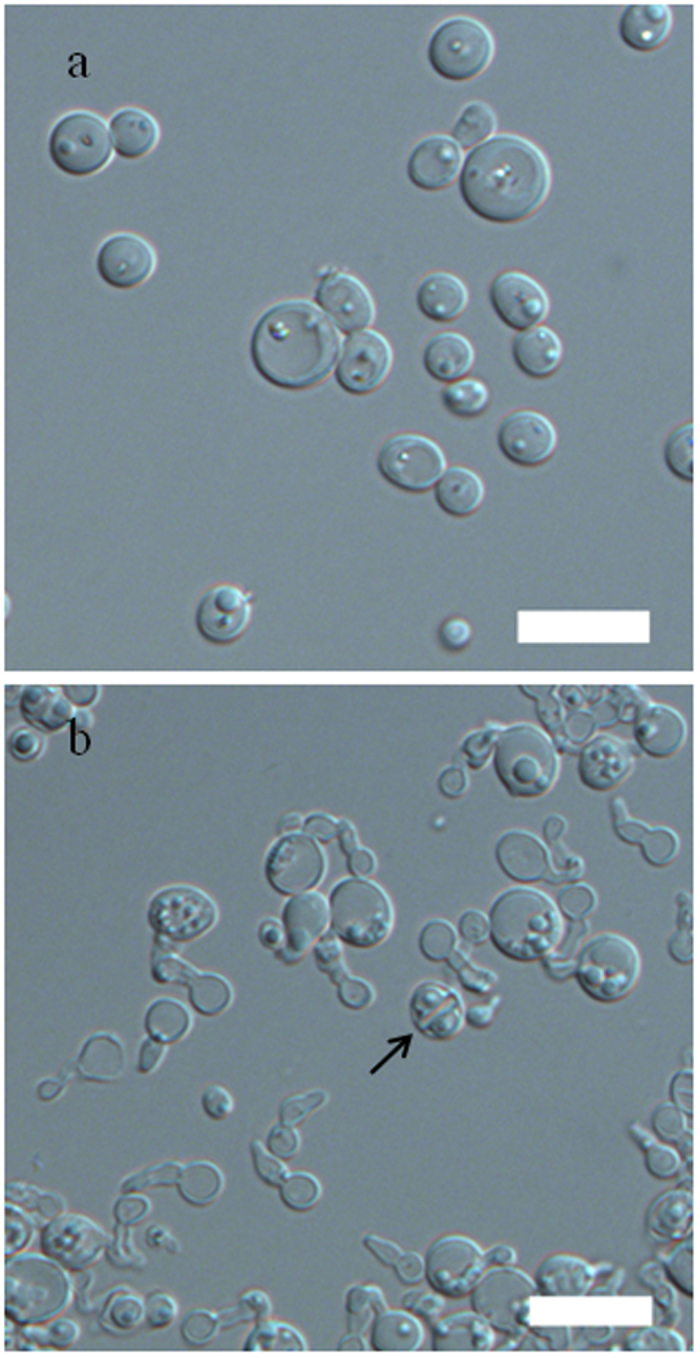
Morphological characterisation of *Scheffersomyces titanus* sp. nov. NYNU 14712^T^. (**a**) Budding cells grown on YM broth for 3 days at 25 °C. (**b**) Ascospores (arrow) formed on corn meal agar after 6 days at 25 °C. Bar, 10 μm.

The new species presents morphological characteristics typical of S. *stipitis*, the type species of the genus *Scheffersomyces*^2^. In addition to cells that reproduced by multilateral budding and formed with pseudohyphae, the novel species produced two hat-shaped ascospores in a deliquescent ascus (Fig. 3). The asci were produced by conjugation between a cell and its bud or between independent cells which are usually observed in its closely related species, such as *S*. *henanensis*, *S*. *segobiensis* and *S*. *stipitis*^2^,^7^. The mode of conjugation suggests the species to be homothallic. Physiologically, this new yeast species is highly similar to its closely related species in the *S*. *stipitis* subclade. However, some phenotypic differences exist between the new species and its closely related species (Table 1). In practice, all of the strains of the new species can be distinguished from *S*. *henanensis*, *S. illinoinensis*, *S*. *segobiensis* and *S*. *stipitis*, their closest phylogenetic relatives, on the basis of sequence comparisons because differences in phenotypic characteristics are minor (Table 1).

### Growth and fermentation of glucose or xylose by the new species

**Figure 4:**
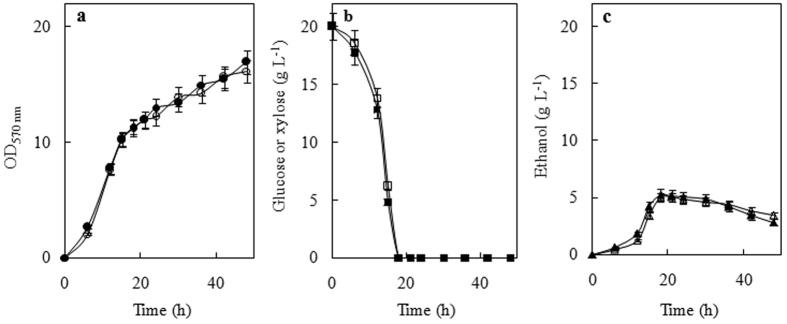
Typical aerobic batch growth of *Scheffersomyces titanus* sp. nov on 20 g L^−1^ of glucose (black symbols) or d-xylose (open symbols). Cell growth (**a**), the consumption of sugars (**b**) and the production of ethanol (**c**) by the strain NYNU 14712^T^ were determined during growth in the rich YP medium.

**Figure 5:**
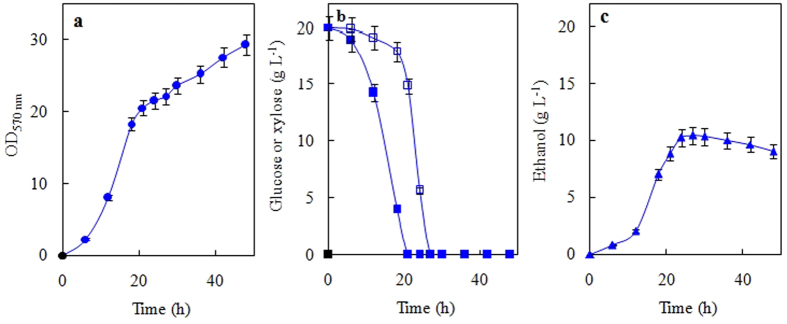
Sugar batch fermentations of *Scheffersomyces titanus* sp. nov on a mixture of 20 g L^−1^ glucose (black symbols) plus 20 g L^−1^ d-xylose (open symbols). Cell growth (**a**), the consumption of sugars (**b**) and the production of ethanol (**c**) by the strain NYNU 14712^T^ were determined during growth in the rich YP medium.

Figure 4 shows the kinetics of growth on 20 g L^−1^ glucose or d-xylose by the strain NYNU 14712^T^. This strain exhibited a typical growth curve where the sugar is efficiently fermented. After the sugar is exhausted from the media, the produced ethanol starts to be consumed and used as a carbon source by the yeast. The strain grew well on both carbon sources and produced practically the same amount of biomass (Fig. 4a). However, the lower levels of ethanol were produced during aerobic growth on glucose or xylose by the strain NYNU 14712^T^ (Y_E/glu_ = 0.28 ± 0.02 g ethanol g^−1^ sugar; Y_E/xyl_ = 0.27 ± 0.03 g ethanol g^−1^ sugar) (Fig. 4c). As found typically for other d-xylose-fermenting yeasts^9^,^10^, this yeast has a clear preference for glucose uptake and fermentation. This characteristic is evident during batch fermentations of a mixture of 20 g L^−1^ glucose plus 20 g L^−1^ d-xylose, where glucose consumption occurs before d-xylose utilization when both sugars are present at the beginning of the fermentation (Fig. 5). Nevertheless, produced ethanol from a mixture of sugars containing glucose and d-xylose (Y_E/sug_ ~ 0.30 g ethanol g^−1^ sugar) at yields similar to those reported for other d-xylose-fermenting *Scheffersomyces* yeasts^14^, *S*. *titanus* may provide a source of genes, enzymes and/or sugar transporters to engineer strains for efficient ethanol production from renewable biomass.

### Taxonomy

Based on their morphology, physiology and the five molecular markers used in this study, the strains above are well supported to represent a distinct taxon, described here as new species in the genus *Scheffersomyces*.

***Scheffersomyces titanus*** F. L. Hui, X. J. Liu & Z. Liu **sp. nov. -**Fig. 3a,b **Fungal Name FN 570250.**

### Etymology

The species name *titanus*; (N. L. gen. n.) refers to the species of the host beetle, *Dorcus titanus*, from which this species was isolated.

#### Description

In YM broth after 3 days at 25 °C, cells are spherical or ovoid (2–6.5 × 2–7 μm) and occur singly or in pairs (Fig. 3a). Budding is multilateral. On YM agar after 3 days at 25 °C, the streak culture is butyrous, white, raised with a smooth surface and has an entire margin. In Dalmau plates after 12 days on cornmeal agar at 25 °C, pseudohyphae are formed, but true hyphae are not formed. On cornmeal agar and 5% malt extract agar after 6 days at 25 °C, conjugated asci are formed and each ascus contains two hat-shaped ascospores. Asci are deliquescent (Fig. 3b). Glucose, galactose (weak), maltose (weak), trehalose and d-xylose (weak) are fermented, but not methyl α-d-glucoside, sucrose, melibiose, lactose, cellobiose, melezitose, raffinose or inulin. Glucose, galactose, l-sorbose (weak), d-glucosamine (weak), d-ribose, d-xylose, d-arabinose, l-rhamnose, sucrose, maltose, trehalose, methyl α-d-glucoside, cellobiose, salicin, arbutin, lactose, melezitose, inulin, soluble starch, glycerol, erythritol, ribitol, xylitol, l-arabinitol, d-glucono1, d-mannitol, d-glucitol, d-Mannitol, *myo*-inositol, 2-keto-d-gluconate, 5-keto-d-gluconate, d-gluconate, succinate and ethanol are assimilated. No growth occurs on l-arabinose, melibiose, raffinose, galactitol, dl-lactate citrate or methanol. Ethylamine, l-lysine and d-tryptophan are assimilated. No growth occurs on nitrate, nitrite, cadaverine, creatine, creatinine, glucosamine or imidazole. Growth occurs at 35 °C, but not at 37 °C. No growth occurs in 10% NaCl plus 5%glucose or in the presence of 1% acetic acid. Growth in 0.1% cycloheximide is positive. Starch-like compounds are not produced. Urea hydrolysis and DBB reactions are absent. The major ubiquinone is Q-9.

#### Specimens examined

Type strain (living and dried) CBS 13926, CICC 33061, NYNU 14712 isolated from *Dorcus titanus* Baotianman Mountain, Henan Province, China 33°27′N, 111°48′E, July 2014 by Y. C. Ren.

## Discussion

In this study, the new d-xylose-fermenting yeast species *S*. *titanus* was described and illustrated based on morphological and molecular characters. Although this new species is highly similar to *S*. *stipitis*-like yeasts in nrDNA sequences, as well as morphology and physiological characteristics (Table 1), the species can be clearly differentiated from its close relatives, *S*. *henanensis*, *S. illinoinensis*, *S*. *segobiensis* and *S*. *stipitis*, by the sequences of *XYL1* and *RPB1* (Figs 1 and 2).

Kurtzman and Robnett (2013) compared the type species of 70 currently recognized genera by sequence divergence in the SSU and LSU rDNAs, *EF*-*1a*, *RPB1* and *RPB2* and found that the genus *Scheffersomyces* is polyphyletic^15^. The results also showed that *S. spartinae* is included in a clade with *Spathaspora passalidarum*, which is distinct from the type species *S*. *stipitis*, although the clade is weakly supported by statistical analyses^15^. Our results from the combined sequence comparison of SSU, ITS, D1/D2 LUS rDNAs and *RPB1* indicated that the genus is not monophyletic; instead, the genus comprises two phylogenetically distinct groups on the tree (Fig. 1). The results strongly suggested that the genus *Scheffersomyces* should be categorised in the monophyletic group near the type species *S*. *stipitis* (Fig. 1). Another group consists of *S*. *gosingicus* and *S*. *spartinae*, which were previously considered as members of *Scheffersomyces*, may become representatives of a novel genus because their phylogenetic relationships within the genus were not supported by bootstrap (Fig. 1).

Three strains of the new species were isolated from the beetles *D. titanus* (Lucanidae, Coleoptera) collected from two different regions in China. The repeated isolation of these yeast strains from *D. titanus* revealed that the species may be involved in a specific yeast-insect association. There are only two previous reports of *Scheffersomyces* species from China. These species include *S*. *gosingica* from forest soil in Taiwan Province^16^ and *S*. *henanensis* from rotten wood in Henan Province^7^. Considering the number of *Scheffersomyces* species found in other countries and the small number of studies on *Scheffersomyces* species found in China, we believed that the discovery of *S*. *titanus* indicates the presence of other species belonging to the genus in this geographic area.

Ethanol production was observed in the fermentation assay. This finding confirmed that the new yeast species can ferment d-xylose to ethanol effectively. Although the molecular basis of efficient xylose fermentation is complex and not yet fully understood^17^,^18^, the discovery and analysis of new *Scheffersomyces* species that can ferment xylose may contribute important genomic information that could be applied to improve the efficiency of pentose assimilation by yeasts^19^, a significant limitation in cellulosic biofuel production. However, further studies have yet to determine whether the novel yeast species can be used directly to produce bioethanol from lignocellulosic hydrolysates, as revealed in *S*. *stipitis*^9^,^10^.

## Methods

### Yeast isolation and culture

Host beetles were collected from two different localities in Henan Province, Central China. The strain NYNU 14712^T^ was isolated from the gut of *D. titanus* (Coleoptera) collected from Baotianman Mountain (33°27′ N and 111°48′ E) in July 2014, and two other strains (NYNU 15875 and NYNU 15860) were found in the gut of the same insect collected from Funiu Mountain (32°45′ N and 113°30′ E) in August 2015. The two mountains were separated from one another by a distance of 50.2 km. The methods used to isolate the yeasts from the gut of insect have been described previously^20^,^21^. The insects were usually placed in Petri dishes for 1–3 days without food prior to dissection. Withholding food aids in eliminating certain contaminating organisms that may be isolated from the gut. The surface was disinfected by submersion in 95% ethanol for 1–2 min. The disinfected surface was then rinsed with 0.7% saline. The insect gut was removed aseptically under a dissecting microscope, and gut segments were streaked on acidified yeast extract–malt extract YM agar (0.3% yeast extract, 0.3% malt extract, 0.5% peptone, 1% glucose and 2% plain agar, adjusted to pH 3.5 with HCl) plates. The plates were then incubated at 25 °C for 3–4 days. The different yeast morphotypes were purified at least twice and stored in YM agar slants at 4 °C and in 15% glycerol at −80 °C.

### Morphological, physiological and biochemical characteristics

Morphological and physiological characteristics were examined in accordance with standard methods employed in yeast taxonomy^22^. Ascospore formation was determined for all new strains by first singly plating then crossing isolates in all possible combinations on YM, McClary’s acetate, cornmeal and 5% malt extract agars. The cultures were incubated at 25 °C and examined weekly by microscopy for up to 28 days. Physiological and biochemical tests were performed by replica plating on solid and in liquid media^22^. Test samples were incubated at 25 °C and results were read weekly for up to 28 days. Ubiquinones were extracted and purified in accordance with the method described by Yamada and Kondo^23^ with slight modifications and determined through HPLC, as described previously^24^.

### DNA amplification and sequencing

Genomic DNA was extracted using an Ezup column yeast genomic DNA purification kit in accordance with the manufacturer’s protocol (Sangon Biotech, Shanghai, China). The concentration, integrity and purity of the total extracted DNA were confirmed through gel electrophoresis in 0.8% agarose in 0.5× Tris-Borate-EDTA (TBE). Nuclear rDNAs for SSU, ITS and D1/D2 LSU were amplified and sequenced, as described previously^25^,^26^,^27^. Two protein-coding genes, namely, *RPB1* and *XYL1*, were amplified using the following degenerate primer pairs: *RPB1*-Af (5′-GARTGYCCDGGDCAYTTYGG-3′) and *RPB1*-Cr (5′-CCNGCDATNTCRTTRTCCATRTA-3′) for *RPB1*^28^,^29^; *XYL1*-forward (5′-GGTYTTYGGMTGYTGGAARSTC-3′) and *XYL1*-reverse (5′-AAWGATTGWGGWCCRAAWGAWGA-3′) for *XYL1*^3^,^5^. The PCR conditions that were recommended in the references for each primer pair were employed. The purified PCR products were sequenced using a Dye terminator cycle sequencing kit (Applied Biosystems, Warrington, USA).

### Phylogenetic analyses

Comparisons with sequences from the international GenBank database (http://www.ncbi.nlm.nih.gov/) were conducted through BLASTN search^30^. Sequences were aligned using the multiple sequence alignment program CLUSTAL X 1.83^31^. Phylogenetic trees were constructed using MEGA software version 5.0^32^. The evolutionary distance data were calculated from Kimura’s two-parameter model^33^ in neighbour-joining analyses^34^. Confidence limits were estimated from bootstrap analysis (1000 replicates)^35^, and only values above 50% were recorded on the resulting trees. The sequences determined from this study, along with reference sequences obtained from GenBank are listed in Table S1.

### Growth conditions and fermentation assays

The cells were grown on the YP medium (1% yeast extract and 2% peptone), adjusted to pH 5.0 with HCl and supplemented with 2% glucose and/or d-xylose. The cells were grown at 28 °C with shaking (160 rpm) in cotton-plugged Erlenmeyer flasks filled with the medium to 1/5 of the volume. The inocula for the growth assays were prepared by aseptically transferring a colony from a plate into 5 mL of glucose or xylose medium; growth was allowed to proceed to stationary phase for 2–3 days. The cells were then inoculated in the fresh media containing similar composition at a rate of 1%. Samples were obtained regularly and centrifuged at 5,000 × *g* for 1 min, and the supernatants were used to determine sugars and ethanol. Glucose and d-xylose levels were determined by HPLC (Waters 410, Milford, MA, USA) as described by Cadete *et al*.^14^. Ethanol was determined with alcohol oxidase (Sigma) and peroxidase (Sangon Biotech, Shanghai, China) as described previously^36^. Growth was followed by turbidity measurements at 570 nm after the medium samples were appropriately diluted in distilled water. The ethanol yields during growth on glucose (Y_E/glu_, g ethanol g^−1^ glucose), xylose (Y_E/xyl_, g ethanol g^−1^ xylose) or glucose plus d-xylose (Y_E/sug,_ g ethanol g^−1^ sugar) were calculated taking into account the amount of sugar consumed at the point of maximum ethanol production. The reported values were the average ± mean deviations obtained from independent duplicate cultures and were analysed using the paired *t*-test.

## Additional Information

**How to cite this article**: Liu, X. *et al*. Taxonomy and physiological characterisation of *Scheffersomyces titanus* sp. nov., a new D-xylose-fermenting yeast species from China. *Sci. Rep.* **6**, 32181; doi: 10.1038/srep32181 (2016).

## Supplementary Material

Supplementary Information

## Figures and Tables

**Table 1:** Physiological characteristics that differentiate *Scheffersomyces titanus* sp. nov. from its related species.

Characteristics	1	2^a^	3^b^	4^c^	5^c^
Fermentation of					
Maltose	w	**+**	**+**	**+**	−
Trehalose	**+**	−	**+**	**+**	**+**
Cellobiose	−	−	−	−	−
Melezitose	−	w	−	−	−
Soluble starch	−	w	−	−	−
Assimilation of					
L-Sorbose	w	−	−	v	**+**
D-Ribose	**+**	−	**+**	**+**	**+**
Melezitose	−	d	**+**	**+**	−
Inulin	**+**	**+**	w	−	−
Soluble starch	**+**	**+**	**+**	**+**	−
Erythritol	**+**	d	**+**	**+**	−
Galactitol	−	**+**	**+**	−	−
D-Gluconate	**+**	**+**	**+**	v	**+**
Growth at 37 °C	−	−	**+**	−	−

The species tested were: 1, *S*. *titanus*; 2, *S*. *henanensis*; 3, *S*. *illinoinensis*; 4, *S*. *stipitis*; 5, *S*. *segobiensis*; +, positive reaction; −, negative reaction; w, weak positive reaction; v, variable reaction.

^a^Data from [7].

^b^Data from [3].

^c^Data from [2].
