# Supplementary material for: Taxonomy and physiological characterisation of Scheffersomyces titanus sp. nov., a new D-xylose-fermenting yeast species from China
Source: Sci Rep. 2016 Aug 25;6:32181. doi: 10.1038/srep32181 (PMC4997322; doi:10.1038/srep32181)
Supplement: Supplementary Information [file srep32181-s1.doc]

**Taxonomy and physiological characterisation of *Scheffersomyces titanus* sp. nov., a new D-xylose-fermenting yeast species from China**

Xiaojing Liu^1^, Wannan Cao^1^, Yongcheng Ren^1^, Longlong Xu^1^, Zehao Yi^1^, Zheng Liu^1^,

Fengli Hui*^1, 2^

*^1^**School of Life Science and Technology, Nanyang Normal University, Nanyang 473061, PR China*

*^2^Henan Provincial Key Laboratory of Funiu Mountain Insect Biology, Nanyang Normal University, Nanyang 473061, PR China*

*Correspondence and requests for materials should be addressed to F. L. H. (email: huifl@126.com or fenglihui@yeah.net)

**Supplementary Information**

**Table S1. GenBank accession numbers of the nucleotide sequences used in this study.**

| Species | Strain no.^1^ | GenBank accession numbers^2^ | | | | |
| --- | --- | --- | --- | --- | --- | --- |
|  |  | SSU | ITS | LSU | *RPB1* | *XYL1* |
| *C*. *tropicalis* | NRRL Y-12968^T^ | EU348785 | AB437068 | U45749 | – | – |
| *L*. *elongisporus* | NRRL YB-4239^T^ | HQ876033 | HQ876042 | HQ876050 | AY653537 | – |
| *S*. *coipomoensis* | NRRL Y-17651^T^ | HQ651931 | HQ652070 | HQ651966 | KC507420 | – |
| *S*. *cryptocercus* | NRRL Y-48824^T^ | JQ714001 | JQ713977 | JQ714021 | JQ713989 | JQ714031 |
| *S*. *ergatensis* | NRRL Y-17652^T^ | AB013524 | EU343826 | U45746 | EU344098 | JQ436926 |
| *S*. *gosingicus* | CBS 11433 ^T^ | HQ876040 | HQ999978 | HQ999955 | – | – |
| *S.* *henanensis* | CBS 12475 ^T^ | JF896577 | HQ127627 | HQ127626 | KF690371 | KF690374 |
| *S*. *insectosa* | NRRL Y-12854^T^ | AB013583 | HQ652064 | U45773 | JN804842 | JQ235697 |
| *S*. *illinoinensis* | NRRL Y-48827^T^ | JN940968 | JN943261 | JN703959 | JN804840 | JQ235694 |
| *S*. *lignosus* | NRRL Y-12856^T^ | HQ651941 | JN943262 | U45772 | JN804837 | JQ235693 |
| *S*. *lignicola* | CBS 10610 ^T^ | AY845351 | HQ652074 | AY845350 | – | – |
| *S.* *parashehatae.* | CBS 12535^T^ | HQ651936 | HQ652051 | HQ651972 | JQ023138 | KC479716 |
| *S*. *queiroziae* | NRRL Y-48722^T^ | – | HM566445 | HM566445 | – | – |
| *S*. *quercinus* | NRRL Y-48825^T^ | JN940981 | JN943260 | JN703957 | JN804838 | JQ008829 |
| *S*. *segobiensis* | NRRL Y-11571^T^ | AB054288 | DQ409166 | U45742 | EF599429 | JQ436925 |
| *S*. *spartinae* | NRRL Y-7322^T^ | FJ153139 | HQ876044 | U45764 | – | – |
| *S*. *shehatae* | NRRL Y-12858^T^ | AB013582 | JN943264 | JQ025409 | JQ436927 | JQ235691 |
| *S*. *stipitis* | NRRL Y-7124^T^ | AB054280 | JN943257 | U45741 | JN804841 | JQ235696 |
| *S. titanus* | CBS 13926 ^T^ | KP054264 | KP054263 | KP054262 | KP054265 | KP054266 |
| *S*. *virginianus* | NRRL Y-48822^T^ | JN940969 | JN943259 | JN703958 | JN804839 | JQ235695 |
| *S. xylosifermentans* | CBS 12540^T^ | HQ876038 | HQ652061 | HQ652020 | JQ023142 | KC479722 |

^1^ CBS, Centraalbureau voor Schimmelcultures, Utrecht, the Netherlands; NRRL Agricultural Research Service Culture Collection, Peoria, Illinois USA; ^T^, type strain.

^2^ SSU, the nearly complete small subunit rDNA; ITS, internal transcribed spacers 1 and 2 together with 5.8S nrDNA; LUS, the D1/D2 domains of the larger subunit rDNA; *RPB1*, RNA polymerase II largest subunit gene; *XYL1* xylose reductase gene.
